# Methods in the Preparation of Teeth

**Published:** 1901-09

**Authors:** Martha Anderson

**Affiliations:** Moline, Ill.


### METHODS IN THE PREPARATION OF TEETH.[1]

BY MARTHA ANDERSON, M.D., MOLINE, ILL.

IN preparing sections of teeth for the microscope it is necessary to proceed in different ways according to the structures desired to be demonstrated. For convenience the methods may be divided into, 1, ground or hard methods; 2, injection methods; 3, structural methods; the structural being subdivided into (*a*) general, or those used to demonstrate cellular structures, and (*b*) special, or those demonstrating nervous tissues.

1. The hard or ground method should be used for studying tooth-tissues *in situ*. The different processes, though differing somewhat in technique, all consist in grinding down sections of teeth thin enough to examine under the microscope and then polishing to a smooth surface and mounting in Canada balsam. They are as follows:

*Nealey's.*—Fresh tissues only should be used, and the method is a rapid one, the sections being ready to mount in thirty minutes. First one side of the tooth and then the other is ground on a dental lathe with a set of emery wheels. The advantages of this method are that the sections do not curl or become brittle.

*White's.*—Sections of teeth are ground between two plates of ground glass with water and pumice powder and finished with ground glass alone. They are mounted in Canada balsam without heat. Or they may be soaked in ether twenty-four hours, transferred to thin collodion, and stained with fuchsin two to three days, when they are hardened in methyl alcohol and ground down at leisure with ground glass and water only.

*Dunkerley's.*—Sections are cut off by means of a thin copper disk fitted to a dental lathe and revolving in a trough containing water and fine corundum powder. They are ground on copper disks and a water-of-Ayr stone.

*Cowardin's.*—From green teeth prepared in turpentine weigh sections are made, then embedded in balsam ground with corundum of four grades successively. They are then polished with the

[1] Read before the American Medical Association, Section on Stomatology, June, 1901.

finest powder—stone lap, dry whiting powder—and the hand, and then mounted in balsam or dry.

*Koch's.*—Objects are soaked in copal solution, dried, hardened, and cemented to a slide, on which they are ground down on a grindstone and on a hone to the requisite thinness, polished, and mounted in balsam.

*Wiel's.*—Tooth is extracted and immediately put into alcohol or aqueous solution of bichloride of mercury, to prevent shrinking. In all manipulations (cutting, sawing, grinding, sectioning) care should be taken that the tooth is at no moment without excess of moisture. The pulp is exposed at both ends and fixed in a saturated solution of bichloride of mercury from eight to twelve hours, and washed in water two hours; then hardened in dilute alcohol, the strength being gradually increased to thirty per cent., eight to twelve hours; fifty per cent., eight to twelve hours; seventy per cent., eight to twelve hours; ninety per cent., to which tincture of iodine one and one-half to two per cent. has been added. Stain in borax-carmine. Wash, dehydrate, clear, embed in balsam, and rub down.

McQuillen attaches sections to a slide with balsam and grinds with a hone.

Latimer grinds on a corundum wheel, an Arkansas wheel, then polishes by rubbing on a smooth and dry plate glass.

*Bödecker's.*—A fresh tooth, or one soaked for a short time in chromic acid, is sliced thin under water and ground as thin as possible upon the corundum wheel of a lathe, always being kept under water. The sections are then placed in chromic acid one-half of one per cent., or saturated aqueous solution of picric acid, to harden tissues and dissolve the lime-salts. They are then stained and mounted.

Hart, after grinding sections thin, immerses them in six per cent. glacial acetic acid, and then treats them in the usual way.

Modifications of these methods have been advised, as those of Smith, Ross, and others.

2. To demonstrate the blood-supply injection methods are necessary. The blood-vessels are filled with colored substances for the purpose of showing their relation to and course through the tissues. Beale's prussian blue is the solution usually used. This mass runs well and has not much tendency to exude from cut vessels. Its formula is as follows:

℞ Price's glycerin,
Tincture sesquichloride of iron,
Potassium ferrocyanide,
Hydrochloric acid (strong),
Distilled water.

Fearnley introduces Carter's carmine into the aorta of an animal, during which time it is kept warm. After the injection it is removed to cold water:

℞ Carmine,
Glacial acetic acid,
Gelatin solution (1 to 6),
Distilled water.

Whitman, in injecting, uses the femoral, or, if that is too small, the carotid, or aorta. The animal is immersed in warm water and normal salt solution forced through the vessels to wash out the blood and dilate the vessels, and then the starch injection mass is forced through.

INJECTION MASS.

| | |
|---|---|
| ℞ Dry starch | 1.00 |
| Chloral hydrate, two and one-half per cent. aqueous solution | 1.00 |
| Alcohol | 0.25 |
| Color | 0.25 |

COLOR.

| | |
|---|---|
| ℞ Dry color (ultra marine) | 1.00 |
| Glycerin | 1.00 |
| Alcohol, ninety-five per cent | 1.00 |

Lepkowsky injects Berlin blue and then hardens the tissues *in situ* in formol, fifty per cent., for two days. Then he decalcifies in nitric acid, ten per cent., from eight to fourteen days, and mounts in celloidin.

Huber used Ehrlich's methylene-blue *intra-vitam* method. After forcing through a normal salt solution, methylene-blue in normal salt solution was injected through the carotid until the lips assumed a deep-blue color. Thirty minutes later the lower jaw was removed and cleansed with a dry cloth. The teeth were removed and the pulp placed on a slide in normal salt solution. In a few moments the characteristic blue color was found to have developed in the axis cylinders of the peripheral nerves. In fresh preparations the axis cylinders stained blue; the other tissues not at all, or faintly. Unfortunately, preparations prepared thus always fade unless a fixative is used promptly. As a fixative a saturated aqueous solution of ammonium picrate (Dogiel) or a solution of ammonium molybdate (Bethe) may be used. The former preparations are mounted in a mixture of glycerole and the ammonium picrate solution; the latter dehydrated and mounted in balsam. Prepared in this way the tissues become so clear that a small pulp may be examined *in toto.* These methods Huber used in a rabbit; they cannot be employed in pulps of human teeth.

With these two methods, the hard and the injection, I have had little experience, having worked mainly with pulps and decalcification of teeth. To demonstrate tissue-structures it is necessary to stain microtome sections with different dyes to bring out the different tissues. Either pulps themselves are used, or decalcification of the hard structures is necessary.

3. *Decalcification Methods.*—Hopewell Smith decalcifies in five per cent chromic acid, or, after hardening in Müller's fluid from three to four weeks, removes to alcohol from ten to twenty days, washes, seals the apical foramen with collodion and places in twelve cubic centimetres of ten per cent. hydrochloric acid for fifteen hours, then adds one and five-tenths cubic centimetres nitric acid, and in forty hours one and five-tenths cubic centimetres nitric acid again. In from seventy-five to eighty hours they are washed in lithium carbonate for a half-hour and cut by freezing method, celloidin or paraffin. If the freezing method is used, they should be dehydrated in thirty per cent., seventy per cent., ninety per cent., and absolute alcohols successively for one minute each to prevent shrinking.

*Lepkowski's Method.*—Pieces one-half millimetre thick are placed in pure formic acid, three pints, and one per cent. aqueous solution gold chloric, six pints, for twenty-four hours; then washed in distilled water and placed in gum arabic and glycerin for twenty-four hours, washed, and embedded in celloidin or paraffin.

KLEINENBERG'S FORMULA.

| | | |
|---|---|---|
| ℞ | Picric acid | 100.00 |
| | Sulphuric acid (strong) | 2.00 |
| | Filter and add distilled water | 300.00 |

EBNER'S FORMULA.

| | | |
|---|---|---|
| ℞ | Hydrochloric acid | 1.00 |
| | Sodium chloride | 10.00 |
| | Distilled water ad | 100.00 |

HAUG'S FORMULA.

| | | |
|---|---|---|
| ℞ | Phloroglucin | 1.00 |
| | Nitric acid | 10.00 |
| | Distilled water | 50.00 |

After this solution tissues stain well and all elements except blood are well preserved.

Schaeffer used a saturated aqueous solution of picric acid containing two per cent. hydrochloric acid and a crystal of picric acid added from time to time. When soft, tissues were placed in alcohol containing lithium carbonate. The alcohol should be changed till no color comes away. Embed in paraffin.

*Squire's Method.*—Hydrochloric acid, 5, glycerin, 95, softens slowly, and does not interfere with structure.

Huber hardens in Müller's fluid for several weeks and then transfers to equal parts of nitric acid, ten per cent., and hydrochloric acid, one per cent.

Bödecker advised chromic acid, one-half to one per cent., used in large quantity and renewed frequently. To enforce the action of the fluid he added a small quantity of dilute hydrochloric acid (one-half drop). This is a good method for softening teeth when still in the jaw. It can be stained with carmine, logwood, or gold chloride. If gold is used, wash well in distilled water to free from alcohol before placing in gold solution (one-half per cent.). Stain one-half to one hour, thoroughly wash in distilled water, and expose to daylight several days.

Lactic acid acts well upon teeth (if diluted sufficiently) by dissolving the lime-salts much faster than the chromic acid. Specimens decalcified in lactic acid are not distinct enough for study with high powers, hence after being softened with lactic acid they must be placed in chromic acid for several weeks.

Boll advises chromic acid, one-thirty-second per cent., and gradually increased in strength until in two weeks it contains two per cent. or over. Decalcify from five to six weeks.

*Hopewell Smith.*—For jaws of embryo mammals: Strip jaws of all tissue except the gums, wash in normal salt solution and place in Müller's fluid for two weeks. Change often, remove to alcohol, and cut by freezing microtome, celloidin, or paraffin. Its advantages are its simplicity, the odontoblasts are of large size, and the formation of the dentinal fibrils are at their highest stage of development. It affects but little the relative positions of dentine, odontoblasts, and pulp.

In using the tooth-pulps alone great care must be exercised in obtaining them that the pulp may be obtained in its entirety. While some workers claim that all that is necessary is to crack open the tooth and lift the loose pulp out, my experience leads me to think differently. Many pulps that I have worked with have had small pieces of dentine attached along the sides. These were unnoticed at first until the knife struck them in microtoming.

In some sections the tomes could be seen extending out from the odontoblasts along the free border of the section; in others these were present only in places, and many were perfectly free from them. Some investigators believe that the nerve-fibrils pass up between the odontoblasts and enter the dental canaliculi side by side with the dentinal fibres. Others differ; but whether so or not, one thing is certain, that there is an intimate connection between the tomes and the dentine, and that, being so delicate, great care is necessary in handlng them. Boll claims that the odontoblasts, by their long processes, which extend into the dentinal canaliculi, are held firmly to the inner surface of the dentine, appearing to the naked eye as a thin, mucus-like film. If this film is scraped off with a knife and brought under the microscope, we see, besides peripheral cells, some parts of the inner pulp-tissue. He therefore advises splitting a tooth and placing pulp and tooth in a very weak solution of chromic acid (one-thirty-second per cent.) for one hour. Remove the loose fragments of tooth and with a thin, sharp knife go between pulp and dentine close to the walls of the pulp-cavity. In specimens obtained thus large quantities of non-medullated nerve-fibres are seen towards the periphery of the pulp, which, when removed in the ordinary way, adhere to the pulp-chamber.

Robertson holds that when a tooth is broken open the odontoblasts cling to the dentine and the pulp remains free, thus proving that the attachment to dentine is stronger.

For ordinary staining, logwood, logwood and eosin, and Van Giessen are good stains; but for special staining, and especially for the nervous tissues which we are so anxious to bring out, special stains are necessary. Of these, the following are largely used: Golgi's stain for demonstrating nerves; Ehrlich's methylene-blue shows axis cylinders of peripheral nerves; Apathy's double stain for differentiating nervous and connective tissues; Mummery's iron and tannin for fine nerve-fibres; Marchi's med. nerve-fibres; Underwood's gold chloride.

For the odontoblasts, place in six per cent. solution of potassium anhydrochromate for twenty-four hours, and tease in picrocarmine. To demonstrate the nerves in the processes of the odontoblasts, Beale crushes a fresh tooth and stains by his method, macerates in strong syrup until soft, examines a few very thin sections, when the acid will be found to render all tissues transparent except the nerves, which are granular.

BIBLIOGRAPHY.

Histology. Notes on Preparation of Teeth. Latham, V. A.
Journal Royal Microscopical Society, 1885, pp. 348.
Journal Royal Microscopical Society, 1890, pp. 529.
Journal Royal Microscopical Society, 1891, pp. 307.
Journal Royal Microscopical Society, 1892, pp. 433.
Dental Microscopy. Hopewell Smith, 1895.
Preparation of Ground Teeth and Bone. S. P. Cowardine. Journal of Applied Microscopy.
Vade Mecum. Lee.
Dental Cosmos, vol. vii. 449; xvi. 121; xx. 652; xxiv. 400; April, 1894, p. 284.
Anal. Hefte, vol. viii., 1897, pp. 568.
Investigations of the Tooth-Pulp. Boll. (Translated by Hirschfeld.) Dental Cosmos, xii. 113.
Study of Odontoblasts. W. G. A. Robertson. Translations Royal Society of Edinburgh.
Innervation of Tooth-Pulp. G. C. Huber. Dental Cosmos, xl. 797.
Beale. How to work with the Microscope. Fourth Edition, 1870, pp. 109-297.
Preparations of Teeth. Latham. Scientific Inquirer, vol. ii. p. 196.
Preparations of Teeth. Latham. Journal Postal Microscopic Society, 1889, p. 134.
Preparations of Teeth. Latham. International Journal of Microscopy, 1892; July, October, 1892; January, 1893, etc.
Bacteriology and Histology. Latham. Dental Register, 1888, p. 477.
Bacteriology and Histology. Latham, 1891, xlv. p. 276.

